# Moderating effects of media literacy and flow on the association between web-based learning environment and knowledge sharing among medical students: a cross-sectional study

**DOI:** 10.3389/fpsyg.2026.1825534

**Published:** 2026-09-28

**Authors:** Chenxi Zhao, Guanjun Wang, Haichen Guo, Libin Yang, Qingfeng Guo

**Affiliations:** 1 Academic Affairs Office, First Affiliated Hospital of Harbin Medical University, Harbin, China; 2 Jinan Hospital of Xuanwu Hospital, Jinan, China; 3 Outpatient Department, First Affiliated Hospital of Harbin Medical University, Harbin, China; 4 Department of Health Management, Harbin Medical University, Harbin, China

**Keywords:** flow, knowledge sharing, media literacy, medical students, web-based learning environment

## Abstract

**Background:**

Online learning has become an integral part of medical education, and understanding the factors associated with medical students’ knowledge sharing is important for designing effective learning environments.

**Objective:**

This study examined the associations among web-based learning environment, media literacy, flow, and knowledge sharing, and tested whether media literacy and flow moderate the association between web-based learning environment and knowledge sharing.

**Methods:**

A cross-sectional online survey was conducted from July to September 2021 in China. Of 1,200 undergraduate medical students invited, 987 valid responses were retained (response rate 82.25%; 70.7% women). Hierarchical linear regression with mean-centered variables was used to examine factors associated with knowledge sharing and to test the moderating effects of flow and media literacy.

**Results:**

The mean knowledge sharing score was 3.91 ± 0.58. Knowledge sharing was positively correlated with web-based learning environment (*r* = 0.360, *p* < 0.01), flow (*r* = 0.565, *p* < 0.01), and media literacy (*r* = 0.460, *p* < 0.01). Flow (*β* = 0.732, *p* = 0.001; Δ*R*^2^ = 0.008) and media literacy (*β* = 0.900, *p* = 0.001; Δ*R*^2^ = 0.009) significantly moderated the association between web-based learning environment and knowledge sharing, although the incremental explained variance was small.

**Conclusion:**

The association between web-based learning environment and knowledge sharing was stronger among medical students with high flow or high media literacy. These findings suggest that improving the web-based learning environment and integrating media literacy education may support knowledge sharing; causal conclusions require longitudinal or interventional evidence.

## Introduction

Knowledge sharing is crucial in innovative learning among college students and is also a key indicator of successful universities (Al-Husseini et al., 2021). The COVID-19 pandemic substantially disrupted face-to-face teaching, and online instruction increasingly replaced traditional classroom formats, creating new opportunities for knowledge sharing (Demuyakor, 2020; Ozamiz-Etxebarria et al., 2021). However, the ‘digital divide’ associated with information asymmetry and unequal access to network resources may also be reinforced, which could hinder knowledge sharing among students.

Knowledge sharing involves the exchange of information, ideas, suggestions, and experiences, and can be understood as the process through which explicit or tacit knowledge is transferred from one person to another student or group of students (Elrehail et al., 2018; Rafique and Anwar, 2019). Medical schools are knowledge-intensive organizations, and the multisource and heterogeneous information enabled by knowledge sharing can broaden medical students’ perspectives. Previous research has shown that knowledge sharing is particularly important for activities that support nurses’ innovation ability in medical education settings (Li-Ying et al., 2016; Wah et al., 2018; Asurakkody and Kim, 2020). Tacit knowledge sharing among doctors, such as sharing clinical experience, skills, or expertise, is associated with the quality of medical diagnosis and decision-making (Henry, 2006; Lee et al., 2019). Interdisciplinary medical teams have also improved communication and knowledge exchange between hospital departments, reducing gaps between specialties (Fronzetti Colladon et al., 2023). As medical knowledge becomes increasingly complex, cross-disciplinary, and comprehensive, social interaction and teamwork are important for helping medical students develop professional skills (San Martín-Rodríguez et al., 2005; Wang and Lin, 2021). At the same time, the concept of ‘private knowledge’ may lead individuals to hoard knowledge to maintain a competitive advantage over peers (Bock and Kim, 2002), which may be unfavorable for both individuals and medical schools. Most previous studies have focused on employees in corporate settings, with less attention to knowledge sharing among knowledge-intensive groups such as medical students. Therefore, exploring knowledge-sharing mechanisms among medical students may help medical school management design interventions that enrich students’ knowledge base and support the quality of medical diagnosis and research.

## Conceptual framework and hypotheses

### Relationship between knowledge sharing and web-based learning environment

Computers and networks provide infrastructure that supports knowledge exchange, and the web-based learning environment can be viewed as the combination of hardware, software, telecommunications, and data communication facilities that support learning (Davenport and Prusak, 1998). In this study, the web-based learning environment refers to the sum of network learning resources, network infrastructure, and learning atmosphere that support medical students’ learning activities. Knowledge sharing may be more effective with the assistance of network technology than in face-to-face interaction alone (Bordia et al., 2006; Buckley et al., 2006). Social media use is a typical example: healthcare professionals, including medical students, are active social media users (Kung and Oh, 2014; Avcı et al., 2015). Cheston et al. (2013) reported that social media tools provide medical students with opportunities for participation and collaborative learning, which may be positively associated with their learning outcomes, for example by integrating collective wisdom to improve professional skills and build confidence in practice (Liu et al., 2020). A well-designed web-based learning environment can also create a discussion atmosphere in which students are willing to express ideas and interact (Mazzolini and Maddison, 2007; Kapur and Kinzer, 2007). Other studies have found that web-based learning environments can provide different resources, educational tools, and collaboration facilities, break time and space constraints, and reduce barriers to knowledge sharing (Karunanayaka, 2008). Therefore, we propose the following hypothesis:

*Hypothesis 1*: Web-based learning environment is positively associated with knowledge sharing.

#### The moderating role of flow

Flow is an important concept in positive psychology. Csikszentmihalyi defined flow as a mental state or experience in which people concentrate on an activity and become fully immersed and energized (Csikszentmihalyi, 1988). Flow is associated with positive outcomes such as higher task performance, a stronger sense of wellbeing, and greater willingness to share (Eisenberger et al., 2005; Tse et al., 2021). Flow can also moderate the strength of associations between variables; previous studies found that flow moderated relations between trusting beliefs, website loyalty, perceived stress, internet addiction, and behavior-related outcomes (Gong et al., 2021; Kim, 2021). Flow has been highly correlated with harmonious web-based learning environments, and there is evidence that flow increases users’ access time to networks (Koufaris, 2002). A successful web-based learning environment allows learners to participate fully in online educational activities and may be positively associated with user behavior on social media (Rodríguez-Ardura and Meseguer-Artola, 2021). In virtual environments, flow mechanisms may support concentration in tasks that involve active participation and social interaction (Lin and Joe, 2012). Higher flow is also associated with stronger willingness to self-disclose (Kwak et al., 2014). Flow may act as a facilitator for extrinsic and intrinsic motivation, and students may share knowledge based on appreciation and helping others (Ahmad and Abdulkarim, 2019; Fu et al., 2017). We therefore formed the following hypothesis:

*Hypothesis 2*: Flow moderates the association between web-based learning environment and knowledge sharing.

#### The moderating role of media literacy

Media literacy education is seen as a potential way to support evidence-based medical education and to improve medical students’ ability to gather, select, and judge information (Corbin et al., 2018). Previous literature suggests that media literacy is associated with people’s behavior, including knowledge sharing (Banerjee and Greene, 2007). Festl found that higher social media literacy was associated with higher participation and interaction behavior among teenagers, including the ability to share knowledge and exchange meaning with others in networks (Festl, 2021). A study of knowledge sharing among library staff showed that media literacy played a completely mediating role between personality factors and knowledge sharing (Keshavarz, 2021). Media literacy can also act as a moderator; Wei et al. indicated that social media literacy moderated the relationship between rational choice factors and fake news sharing (Wei et al., 2023). Media literacy is closely related to internet use. Grover et al. noted that poor media literacy may reduce students’ opportunities to view case histories and investigations at their convenience, to discuss among themselves, and to share internet-based information, links, and e-book pages (Grover et al., 2020). Such limitations may hinder learners from interacting in the network environment and thus affect knowledge sharing. We therefore propose the following hypothesis:

*Hypothesis 3*: Media literacy moderates the association between web-based learning environment and knowledge sharing.

We examined the following three hypotheses in this study (Figure 1).

**Figure 1:**
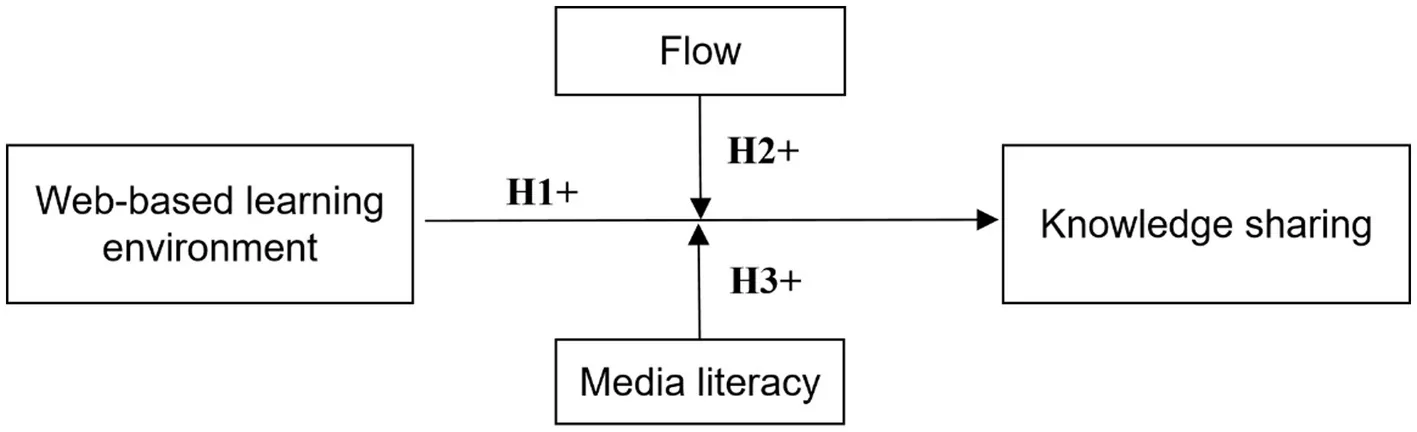
Hypothesized model.

## Methods

### Study design, participants, and ethics

Considering the time-effectiveness, flexibility, cost-effectiveness, and accessibility of online surveys (Chang, 2013), we conducted a cross-sectional study in Heilongjiang Province, China, from July to September 2021. This study involved human participants. It was performed in accordance with the Declaration of Helsinki and was approved by the Ethics Committee of the Institutional Review Board of Harbin Medical University. Electronic informed consent was obtained from all respondents, who participated voluntarily. All collected data were anonymized and kept confidential to protect participants’ privacy.

Undergraduate medical students from Harbin Medical University and Jiamusi University were recruited using a combination of cluster sampling and stratified random sampling. To make the procedure transparent, students were first stratified by university and grade. Within each stratum, classes were treated as clusters and were randomly selected. All students in the selected classes were then invited to participate, and those who met the inclusion criteria and completed the questionnaire were retained. This approach balanced broad grade-level coverage with the practical constraints of administering supervised online surveys during the pandemic. Although a minimum of 100 participants is sometimes suggested for basic regression analyses (Mat Halif et al., 2020), detecting interaction effects requires substantially larger samples. We therefore determined the sample size *a priori*: for hierarchical regression with up to eight predictors, approximately 800 participants were required to detect a small interaction effect (*f*^2^ = 0.01, *α* = 0.05, powe*r* = 0.80) (Cohen, 1988; Faul et al., 2009). The final sample of 987 exceeded this requirement.

Given the constraints imposed by the COVID-19 pandemic, we administered the survey electronically via Questionnaire Star, a widely used online survey platform that enabled real-time response monitoring. The electronic questionnaires were distributed to students’ mobile phones, primarily during class breaks, and each IP address was restricted to a single submission to avoid duplicate responses. In total, 987 valid questionnaires were retained (effective rate 82.25%), after excluding incomplete responses and responses that failed the quality control questions. Inclusion criteria were: (1) undergraduate medical students; (2) voluntary participation with electronic informed consent; and (3) serious and complete responses.

### Measures

#### Measurement of knowledge sharing

Knowledge sharing was measured using the Chinese version of the Knowledge Sharing Scale adapted by Wang Juanru from the Intention to Share Knowledge Scale developed by Bock and Kim (2002). This scale focuses on the extent and frequency of knowledge-sharing behaviors and has been used and validated in Chinese populations, with good reliability and cross-cultural adaptability in previous studies (Qiang et al., 2022). It comprises three items scored on a 5-point Likert scale from 1 (strongly disagree) to 5 (strongly agree); higher scores indicate more frequent knowledge sharing. In this study, Cronbach’s α was 0.809.

#### Measurement of web-based learning environment

The web-based learning environment was measured with five items adapted from the scale developed by Li (2010), which was originally based on the framework proposed by Thurmond et al. (2002) to measure the use of technology in nursing higher education. Each item is scored from 1 (strongly disagree) to 5 (strongly agree), and the total score ranges from 5 to 25, with higher scores indicating a more supportive web-based learning environment. In this study, Cronbach’s *α* was 0.860 (Gliem and Gliem, 2003).

#### Measurement of flow

Flow was measured with four items adapted from Chang and Zhu (2012), who validated the scale for assessing users’ continuance intention to use social networking sites in China; the original scale was developed by Lee and Tsai (2010). The scale has shown high reliability and validity in Chinese samples. Each item is scored from 1 (strongly disagree) to 5 (strongly agree), with total scores ranging from 1 to 20; higher scores indicate greater flow. In this study, Cronbach’s α was 0.807.

#### Measurement of media literacy

Media literacy was measured with the four-item scale developed by Ma (2020), which was designed for the Chinese context and assesses individuals’ ability to access, evaluate, and use media information. Example items include ‘I can well identify bad content on media (mobile phones, computers, and other information carriers)’ and ‘Usually I can use the media (mobile phone, computer, and other information carriers) to get information as quickly as possible.’ Items are scored from 1 (strongly disagree) to 5 (strongly agree); total scores range from 1 to 20, with higher scores indicating higher media literacy. In this study, Cronbach’s α was 0.886.

#### Measurement of participants’ personal characteristics

Seven demographic variables were collected: gender, grade, one-child status, origin of student, parenting style, academic performance, and experience of student leadership. ‘Origin of student’ refers to the place of registered residence before enrollment, classified as ‘urban’ or ‘non-urban.’ ‘Academic performance’ was divided into five levels: top, above average, average, below average, and low. ‘Experience of student leadership’ was coded as ‘yes’ or ‘no’ according to whether the student was a class leader or had joined an autonomous management organization such as the student union. Parenting style was based on Maccoby and Martin’s framework (Maccoby and Martin, 1983): authoritative (high demands and high support), authoritarian (high demands and low support), permissive (low demands and high support), and uninvolved (low demands and low support). Gender, origin of student, and one-child status were dichotomous variables and were compared using *t*-tests; grade, parenting style, academic performance, and student leadership were compared using one-way ANOVA.

### Statistical analysis

In the model, the web-based learning environment was the predictor variable, flow and media literacy were the moderators, and knowledge sharing was the outcome. Mean knowledge-sharing scores across demographic groups were compared using independent-samples *t*-tests or one-way ANOVA, and Pearson correlation was used to examine bivariate associations among web-based learning environment, flow, media literacy, and knowledge sharing. Hierarchical multiple regression was used to test whether a quantitative variable moderates the association between two other quantitative variables (Baron and Kenny, 1986). Before the analyses, continuous predictor and moderator variables were mean-centered, and interaction terms were created as products of the centered variables. Categorical control variables were dummy-coded, with the first category of each variable serving as the reference category.

Demographic control variables were entered in Model 1; web-based learning environment and flow were added in Model 2, and their interaction term in Model 3; web-based learning environment and media literacy were added in Model 4, and their interaction term in Model 5. A significant interaction term was interpreted as supporting the corresponding moderation hypothesis. Models were estimated with the PROCESS macro in IBM SPSS 26.0, and simple slope analyses at ±1 SD of each moderator were used to visualize significant interactions. All analyses were performed in IBM SPSS 26.0 with two-tailed *p* < 0.05 considered statistically significant. Regression assumptions were checked as follows: linearity was assessed with partial regression plots; residuals were approximately normally distributed; homoscedasticity was evaluated with residual plots; and influential observations were examined using Cook’s distance (all values below 1). Multicollinearity was assessed with the variance inflation factor (VIF); across all predictors in Models 1–5, VIF values ranged from 1.01 to 1.32, all below the conventional threshold of 10.

## Results

### Descriptive results

A total of 987 medical students were included: 698 (70.7%) women and 289 (29.3%) men; 560 (56.7%) from non-urban areas; 548 (55.5%) only children; 626 (63.4%) reporting a permissive parenting style; 347 (35.2%) reporting average academic performance and 331 (33.5%) above-average performance; and 473 (47.9%) with no experience of student leadership (Table 1).

**Table 1:** Characteristics of participants and univariate associations with knowledge sharing (*N* = 987).

Characteristics	*n* (%)	Knowledge sharing (M ± SD)	Test statistic (*t*/*F*)	*p*	Effect size
Gender			*t* = 2.05	0.04	*d* = 0.134
Male	289 (29.3)	3.97 ± 0.65			
Female	698 (70.7)	3.89 ± 0.54			
Grade			*F* = 1.427	0.22	*η*^2^ = 0.006
Freshman	326 (33.0)	3.93 ± 0.58			
Sophomore	302 (30.6)	3.95 ± 0.59			
Junior	185 (18.7)	3.83 ± 0.59			
Senior	163 (16.5)	3.90 ± 0.53			
Senior 5 and above	10 (1.0)	3.83 ± 0.61			
Origin of student			*t* = 3.09	0.002	*d* = −0.191
Urban	427 (43.3)	3.85 ± 0.57			
Non-urban	560 (56.7)	3.96 ± 0.58			
Whether they are the only child in their family			*t* = 2.97	0.003	*d* = 0.191
Yes	548 (55.5)	3.96 ± 0.60			
No	439 (44.5)	3.85 ± 0.55			
Parenting style			*F* = 6.35	< 0.001	*η*^2^ = 0.019
Uninvolved	106 (10.7)	3.82 ± 0.54			
Permissive	626 (63.4)	3.91 ± 0.58			
Authoritarian	90 (9.1)	3.78 ± 0.58			
Authoritative	165 (16.7)	4.06 ± 0.58			
Academic performance			*F* = 8.12	<0.001	*η*^2^ = 0.032
Top	141 (14.3)	4.03 ± 0.59			
Above average	331 (33.5)	4.01 ± 0.58			
Average	347 (35.2)	3.84 ± 0.55			
Below average	121 (12.3)	3.85 ± 0.58			
Low	45 (4.6)	3.64 ± 0.62			
Experience of student leadership			*F* = 1.834	0.14	*η*^2^ = 0.006
Class leader	116 (11.8)	3.91 ± 0.59			
Student union leader	289 (29.3)	3.87 ± 0.56			
Both class leader and student union leader	109 (11.0)	4.02 ± 0.51			
Not a student leader	473 (47.9)	3.92 ± 0.60			

^a^Effect size is Cohen’s d for *t*-tests and eta-squared for one-way ANOVA.

### Knowledge sharing status and correlations among continuous variables

The mean knowledge sharing score was 3.91 ± 0.58. Web-based learning environment (*r* = 0.360, *p* < 0.01), flow (*r* = 0.565, *p* < 0.01), and media literacy (*r* = 0.460, *p* < 0.01) were positively correlated with knowledge sharing. Web-based learning environment was also positively correlated with flow (*r* = 0.396, *p* < 0.01) and media literacy (*r* = 0.299, *p* < 0.01), and flow was positively correlated with media literacy (*r* = 0.514, *p* < 0.01) (Table 2).

**Table 2:** Correlations among continuous variables (*N* = 987).

Variables	Mean ± SD	1	2	3	4
Knowledge sharing	3.91 ± 0.58	1			
Web-based learning environment	3.55 ± 0.74	0.360^**^	1		
Flow	3.70 ± 0.60	0.565^**^	0.396^**^	1	
Media literacy	4.15 ± 0.57	0.460^**^	0.299^**^	0.514^**^	1

SD, standard deviation. **p < 0.01.

### Hierarchical linear regression analysis

The results of the hierarchical linear regression are shown in Table 3. Model 1 included demographic control variables (gender, origin of student, one-child status, academic performance, and parenting style) to reduce potential confounding. Model 2 added web-based learning environment and flow. Web-based learning environment was significantly positively associated with knowledge sharing (*β* = 0.159, *p* < 0.001), supporting Hypothesis 1, and flow was also positively associated with knowledge sharing (*β* = 0.480, *p* < 0.001). Together these variables improved model fit (adjusted *R*^2^ = 0.352, Δ*R*^2^ = 0.305, *p* < 0.001). In Model 3, the web-based learning environment × flow interaction was significantly associated with knowledge sharing (*β* = 0.732, *p* = 0.001), supporting Hypothesis 2. Simple slope analysis indicated that the positive association between web-based learning environment and knowledge sharing was not statistically significant at low flow (*B* = 0.051, SE = 0.030, *t* = 1.68, *p* = 0.094, 95% CI [−0.009, 0.111]) but was statistically significant at high flow (*B* = 0.178, SE = 0.026, *t* = 6.80, *p* < 0.001, 95% CI [0.127, 0.229]; Figure 2).

**Table 3:** Results of hierarchical linear regression analysis (*N* = 987).

Variable	Knowledge sharing				
	M_1_*(β)*	M_2_*(β)*	M_3_*(β)*	M_4_*(β)*	M_5_*(β)*
Control variables					
Gender	−0.078^*^	−0.057^*^	−0.053^*^	−0.061^*^	−0.058^*^
Origin of student	0.067	0.029	0.032	0.016	0.019
Academic performance	0.171^***^	−0.097^***^	−0.097^***^	−0.120^***^	−0.124^***^
The one-child	−0.049	−0.013	0.006	−0.038	−0.030
Parenting style	0.080^**^	0.042	0.037	0.011	0.014
Predictor variable					
Web-based learning environment		0.159^***^	−0.328^*^	0.241^***^	−0.434^*^
Moderator variable					
Flow		0.480^***^	0.102		
Media literacy				0.366^***^	−0.053
Interaction					
Web-based learning environment * Flow			0.732^**^		
Web-based learning environment * Media literacy					0.900^**^
*F*	10.774^***^	231.260^***^	11.754^**^	159.830^***^	11.778^**^
*Adjusted R^2^*	0.047	0.352	0.359	0.281	0.289
*∆R^2^*	0.052	0.305	0.008	0.234	0.009

^a^*p < 0.05; **p < 0.01; ***p < 0.001. *β* is the standardized regression coefficient. Continuous predictors and moderators were mean-centered before interaction terms were computed.

^b^Model 1: demographic variables only; Models 2 and 4: main effects; Models 3 and 5: main effects plus interaction.

**Figure 2:**
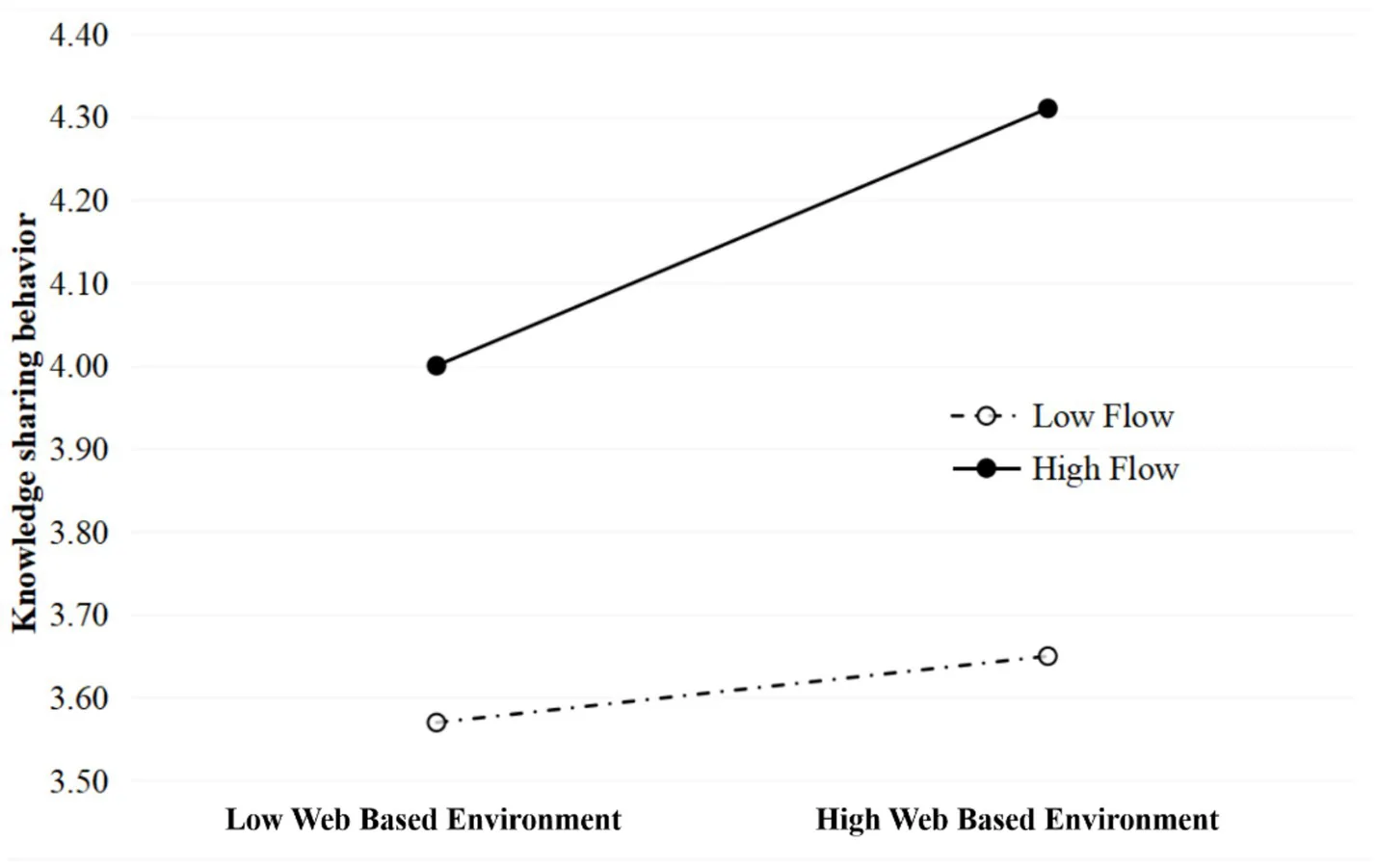
Simple slopes for the association between web-based learning environment and knowledge sharing at high and low flow.

In Model 4, web-based learning environment (*β* = 0.241, *p* < 0.001) and media literacy (*β* = 0.366, *p* < 0.001) were each positively associated with knowledge sharing, and model fit improved (adjusted *R*^2^ = 0.281, Δ*R*^2^ = 0.234, *p* < 0.001). In Model 5, the web-based learning environment × media literacy interaction was significantly associated with knowledge sharing (*β* = 0.900, *p* = 0.001), supporting Hypothesis 2. Simple slope analysis indicated that the positive association between web-based learning environment and knowledge sharing was statistically significant at both low media literacy (*B* = 0.102, SE = 0.035, *t* = 2.89, *p* = 0.004, 95% CI [0.033, 0.171]) and high media literacy (*B* = 0.235, SE = 0.026, *t* = 9.07, *p* < 0.001, 95% CI [0.185, 0.287]; Figure 3). Although both interaction terms were statistically significant, their incremental explained variance was small (Δ*R*^2^ = 0.008 and 0.009), indicating that the practical magnitude of these moderation effects should be interpreted cautiously.

**Figure 3:**
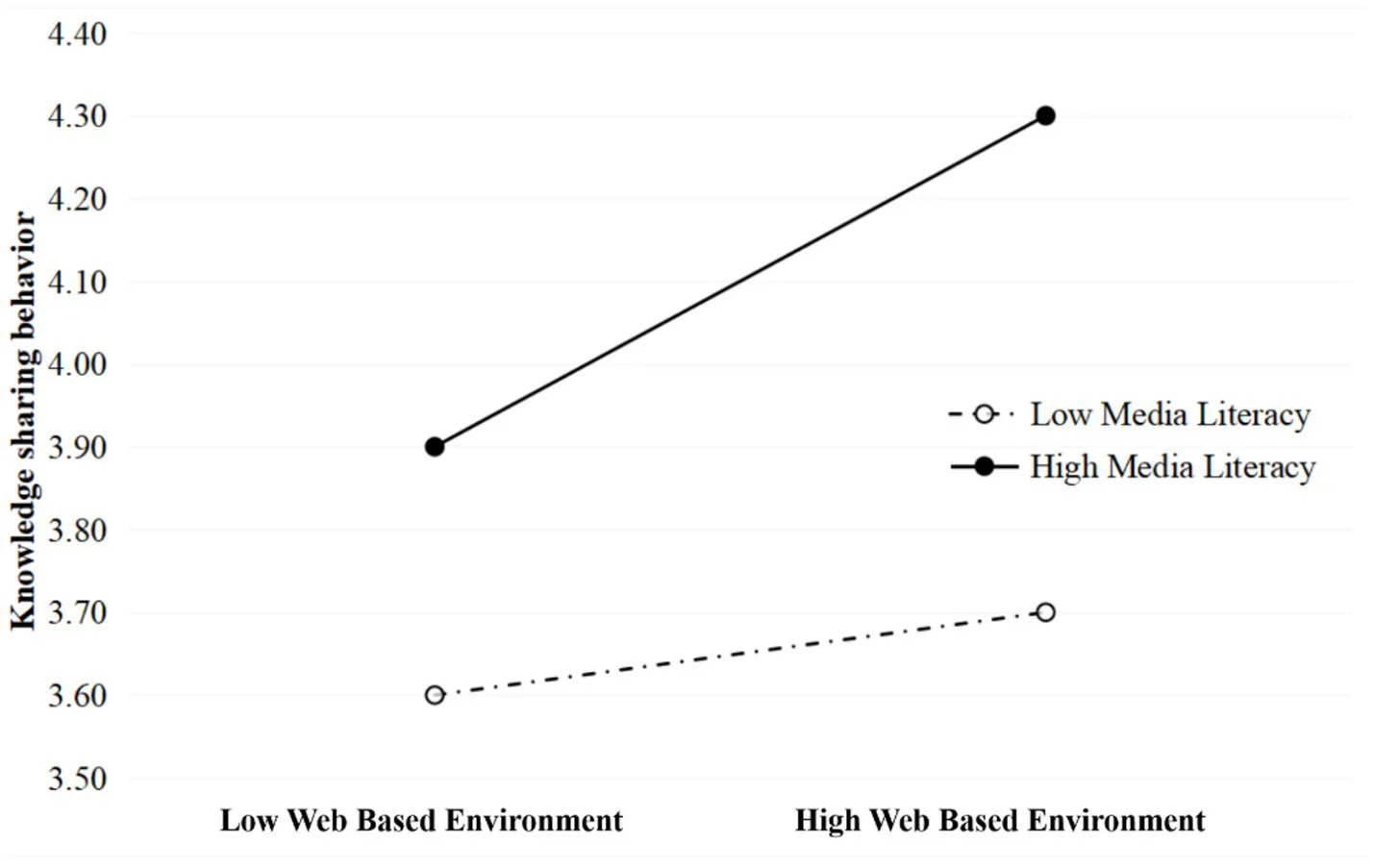
Simple slopes for the association between web-based learning environment and knowledge sharing at high and low media literacy.

## Discussion

### Current status of knowledge sharing and difference in levels of participation in knowledge sharing across various participants’ characteristics among Chinese medical students

The mean knowledge sharing score (3.91 ± 0.58) was above the theoretical midpoint of 3 (Fisher and Marshall, 2009), suggesting that the medical students in this sample generally reported relatively frequent knowledge-sharing behavior. One possible interpretation is that collectivist cultural values, which emphasize group harmony and interdependence, may support cooperative behaviors such as knowledge sharing (Alavi et al., 2005; Nguyen et al., 2022); the pandemic may have further intensified collectivistic expression in China (Han et al., 2021; Gong et al., 2021). Because cultural values were not directly measured in this study, this interpretation should be regarded as speculative.

Several demographic characteristics were associated with knowledge sharing, although the effect sizes were generally small. Male students reported slightly higher knowledge sharing than female students, consistent with earlier findings that male learners may be more eager to establish relationships in online environments and therefore engage more actively in online knowledge sharing (Ma and Yuen, 2011). Non-urban students reported higher scores than urban students. One possible explanation is that social class may shape individuals’ interpersonal orientations (Kraus et al., 2012), and students from non-urban backgrounds may be more attentive to others and more prosocial in their interactions (Miller et al., 2015). However, the urban/non-urban distinction was based on a single binary self-report item, and it cannot support strong claims about egoistic or prosocial social cognition; future studies should assess these constructs directly. Students with higher academic performance reported higher knowledge sharing, possibly because those with stronger academic records have higher self-efficacy and participate more actively in learning activities (Yilmaz, 2016). Authoritative parenting was associated with higher knowledge sharing than other parenting styles, which may reflect the protective and supportive features of this style (Ruiz Hernández et al., 2018); nevertheless, parenting style was not measured longitudinally, so this association should be interpreted cautiously. Only-child students reported higher knowledge sharing than non-only children, which contrasts with the stereotype of only children as selfish (Polit et al., 1980); this may relate to higher agreeableness associated with only-child status (Jia et al., 2022), but this explanation also requires direct measurement in future research.

### The positive relationship between web-based learning environment and knowledge sharing among Chinese medical students

The results showed that a more supportive web-based learning environment was positively associated with knowledge sharing, which is consistent with the view that knowledge exchange depends on accessible infrastructure, resources, and a conducive online atmosphere (Mazzolini and Maddison, 2007; Kapur and Kinzer, 2007; Karunanayaka, 2008). During the pandemic, medical students spent more time learning online, and the shift to virtual spaces created new opportunities for peer interaction and knowledge exchange. A well-designed online learning environment may reduce barriers to communication, support equal dialogue, and facilitate the flow of information among students (Miller et al., 2015). These findings suggest that investment in network infrastructure and user-friendly online platforms may support knowledge sharing; however, causal conclusions require longitudinal or interventional evidence.

### Moderating effect of flow between web-based learning environment and knowledge sharing among Chinese medical students

Flow significantly moderated the association between web-based learning environment and knowledge sharing. Simple slope analysis indicated that the association was stronger among students with high flow than among those with low flow. Students who experience flow in online learning may be more deeply engaged, perceive greater control over the learning environment, and be more motivated to participate in knowledge exchange (Mochklas et al., 2022; Csikszentmihalyi and Rathunde, 1992). By contrast, students with low flow may be less immersed and less motivated to engage in online activities. Practically, designing fluid online learning experiences and helping students set achievable goals may support both flow and knowledge sharing. Because the incremental variance explained by the interaction was small, these implications should be viewed as preliminary.

### Moderating effect of media literacy between web-based learning environment and knowledge sharing

Media literacy was positively associated with knowledge sharing and significantly moderated the association between web-based learning environment and knowledge sharing, with a stronger association at high levels of media literacy. Students with higher media literacy may be better able to locate, evaluate, and use online information, which may increase their confidence and motivation to share knowledge with peers (Lee et al., 2023). Students with lower media literacy may be less engaged in online networks and therefore participate less in knowledge exchange (Buente and Robbin, 2008). These possibilities should be tested directly in future research. In educational practice, integrating media literacy training into the medical curriculum may help students use online resources more effectively and participate more fully in knowledge-sharing activities.

## Conclusion and limitations

In summary, a more supportive web-based learning environment was positively associated with knowledge sharing among Chinese medical students, and this association was stronger among students with higher flow or higher media literacy. The significant interaction effects, however, explained only a small amount of additional variance, and the cross-sectional self-report design precludes causal conclusions. For medical educators, these findings suggest potentially useful directions rather than confirmed interventions: strengthening network infrastructure and optimizing online learning platforms, supporting students in achieving flow by matching task difficulty to skill, and integrating media literacy education into the curriculum. Longitudinal or interventional studies are needed to determine whether changes in these factors lead to higher knowledge sharing.

This study has several limitations. First, the cross-sectional design and single time point mean that the results support associations rather than causal relationships; longitudinal or experimental designs are needed to establish direction and causality. Second, all variables were self-reported and collected at the same time through the same online platform, which makes the study vulnerable to common method bias and social desirability bias; future studies should use temporal separation, multi-source data, or a formal common-method-variance test (Podsakoff et al., 2003). Third, although the sampling procedure combined cluster and stratified random sampling and the sample was large, participants came from two universities in Heilongjiang Province, and online learning behavior during the COVID-19 pandemic may have been unusual; the findings may not generalize to other regions, countries, disciplines, or post-pandemic educational settings. Fourth, the knowledge sharing scale included only three items, which may not fully capture the complexity of knowledge-sharing behavior, and we did not conduct confirmatory factor analysis in this study; future research should evaluate the factorial validity of the scales in Chinese medical student populations.

## Data Availability

The raw data supporting the conclusions of this article will be made available by the authors, without undue reservation.
